# Effect of Metformin on Clinical Course of Non-Diabetic Patients with Ischemic Stroke: Metformin and Outcomes in Non-diabetic Ischemic Stroke

**DOI:** 10.31661/gmj.v14i.4049

**Published:** 2025-11-06

**Authors:** Behnaz Behbudi, Mohsen Ebrahimimonfared, Alireza Kamali, Somayeh Nikfar, Ramin Parvizrad

**Affiliations:** ^1^ Department of Emergency Medicine, Guilan University of Medical Sciences, Rasht, Iran; ^2^ Department of Neurology, School of Medicine, Arak University of Medical Sciences, Arak, Iran; ^3^ Department of Anesthesiology and Critical Care, School of Medicine, Arak University of Medical Sciences, Arak, Iran; ^4^ Department of Gynecology, Arak University of Medical Sciences, Arak, Iran; ^5^ Department of Emergency Medicine, Arak University of Medical Sciences, Arak, Iran

**Keywords:** Metformin, Ischemic Stroke, Non-diabetic, NIHSS

## Abstract

**Background:**

Background: Metformin is commonly used in diabetic patients, but its neuroprotective effects in non-diabetic stroke patients are less understood. This study aimed to evaluate the effect of metformin on the clinical course of acute ischemic stroke in non-diabetic patients.

**Materials and Methods:**

Materials and Methods: In this double-blind randomized clinical trial, 70 non-diabetic patients with acute ischemic stroke confirmed by brain imaging (CT or MRI) within 24 hours of symptom onset were randomly assigned to receive either metformin (500 mg once daily) or placebo for three months, alongside standard care. NIHSS sub-scores were categorized into clinically relevant groups (0=no deficit, 1=mild, 2=moderate, 3=severe) to account for heterogeneity of stroke manifestations. Clinical outcomes, including motor, sensory, visual, and facial function, were assessed at baseline, one, two, and three months. Adverse events were monitored throughout the study.

**Results:**

Results: No serious adverse events were observed; mild gastrointestinal symptoms occurred in 2 patients (5.7%) in the metformin group. Compared with placebo, metformin significantly improved overall NIHSS scores at two and three months (P=0.021 and P=0.003), with notable improvements in motor, sensory, facial, and visual functions. Best Gaze remained normal in most patients. These findings are consistent with previous RCTs reporting neuroprotective effects of metformin in non-diabetic stroke patients.

**Conclusion:**

Conclusion: Metformin at 500 mg daily for three months is well tolerated and significantly improves neurological outcomes in non-diabetic patients with acute ischemic stroke, particularly in motor, sensory, facial, and visual domains. These results support the potential use of metformin as an adjunct therapy in stroke rehabilitation.

## Introduction

Ischemic stroke is one of the most common and important cerebrovascular diseases that leads to acute neurological deficits by blocking blood flow in certain areas of the brain [1][2]. The disease not only imposes a significant burden on public health systems, but is also a leading cause of disability and premature death worldwide [3]. Despite advances in acute treatments such as thrombolysis and thrombectomy, the long-term prognosis of many patients remains unfavourable, especially in populations without classic underlying diseases such as diabetes [4][5]. Therefore, the identification of pharmacological agents with neuroprotective potential in the subacute and chronic stages of stroke is a scientific and clinical imperative. Metformin, a biguanide drug commonly used in the treatment of type 2 diabetes, has attracted much attention in recent years as an agent with multiple effects beyond glycemic control. In fact, in diabetic patients, glycemic control with metformin before stroke is associated with reduced neurological severity and improved acute-phase outcomes due to activation of adenosine monophosphate-activated protein kinase (AMPK) [6][7][8].

Several clinical and preclinical studies have suggested that metformin may improve neurological outcomes after ischemic stroke. For instance, Abbasi et al. (2018) reported reduced NIHSS scores in non-diabetic stroke patients receiving metformin, while animal studies have demonstrated enhanced neurogenesis, angiogenesis, and neuroplasticity [9].

In other words, metformin can prevent or reduce neuronal damage caused by cerebral ischemia by activating the AMPK (AMP-activated protein kinase) pathway and modulating inflammatory processes, oxidative stress, and mitochondrial function. Also, some data suggest that metformin may play an important role in increasing neurogenesis, angiogenesis, and recovery of neurological functions [9].

In a study, it was found that ischemic lesions in rats with chronic kidney disease (CKD), which are larger and more inflammatory, showed lower AMPK activity than those in rats with normal renal function, suggesting a causal relationship between AMPK activity and stroke severity in CKD [10]. However, clinical evidence on the effect of metformin in nondiabetic patients with ischemic stroke is limited and inconsistent. Some studies have shown that metformin administration before stroke can reduce the severity of brain damage, while others have reached different results [11][12]. These inconsistencies may be due to differences in study design, study population, and assessment methods. Given the potential of metformin in improving neurological outcomes and the gap in clinical studies, the present study was designed to investigate the effect of metformin administration on the clinical course of nondiabetic patients with ischemic stroke.

## Materials and Methods

### Study Type and Population

This study was designed and conducted as a randomized, double-blind clinical trial at Arak University of Medical Sciences and Health Services. The study population included all non-diabetic patients over 18 years of age with no known cardiovascular disease and a first ischemic stroke who had been admitted to the emergency department of Hazrat-e-Vali-e-Asr and Amir-al-momenin hospitals in Arak within the first 24 hours of symptoms.

### Sampling Method and Sample Size

Sampling was done in an accessible manner, and all patients were randomly divided into two treatment groups. The sample size was calculated based on the following formula. The number of subjects was calculated as 80, which was reduced to 70 according to the inclusion and exclusion criteria.


n = \frac{(Z_{1-\alpha/2} + Z_{1-\beta})^{2} \times \left[\frac{\delta_{1} + \delta_{2}}{2}\right]^{2}}{(\mu_{1} - \mu_{2})^{2}}

Z_{1-\alpha/2} = 1.96

Z_{1-\beta} = 2.33

\mu_{1} = 6.9

\mu_{2} = 4.4

\delta_{1} = 3.6

\delta_{2} = 3.3

\alpha = 0.05

\beta = 0.2

### Inclusion and Exclusion Criteria

Inclusion criteria included non-diabetic patients over 18 years of age with a first ischemic stroke who had been admitted to the hospital within the first 24 hours of symptoms and had no established cardiovascular disease. Patients with renal dysfunction (creatinine levels greater than 1.4 mg/dl in women and greater than 1.5 mg/dl in men), contraindications or sensitivity to metformin, non-ischemic stroke, established heart disease, with drug addiction or abuse, and patients receiving fibrinolytic therapy were excluded from the study.

### Data Collection Tool

Data were collected using the NIHSS checklist, which consisted of two sections: demographic characteristics and clinical data.

To account for baseline differences in Visual Field scores, all subsequent analyses were adjusted using ANCOVA with baseline value as a covariate. This approach ensures that the observed treatment effects reflect the intervention rather than pre-existing differences.

For symptom-specific analysis, NIHSS sub-scores were categorized into clinically relevant groups: 0=no deficit, 1=mild deficit, 2=moderate deficit, 3=severe deficit. This approach allows for accurate assessment of changes in patients presenting with specific deficits. Neurological deficits were assessed using the National Institutes of Health Stroke Scale (NIHSS), a validated clinician-administered tool widely used for quantifying stroke severity across multiple neurological domains, which indicates the appropriate validity of the questionnaire.

### Methods

After approval of the project by the Research Centre and Ethics Committee of the Arak University of Medical Sciences and Health Services (under the ethics code of IR.ARAK.MU.REC.1395.137) and was registered at the Iranian Registry of Clinical Trials (IRCT) under the registration number IRCT20141209020258N73, patients were randomly divided into two groups of treatment and control. Both groups underwent standard treatment (heparin - clopidogrel - atorvastatin and aspirin) and the same physiotherapy. In the first group, metformin was administered at a dose of 500 mg once a day after breakfast for 3 months, starting 24 hours after the onset of symptoms; and placebo, a tablet made from wheat flour and completely similar to metformin, was administered once a day after breakfast for 3 months, starting 24 hours after the onset of symptoms.

Diagnosis of acute ischemic stroke was confirmed using brain imaging (CT scan or MRI) performed within 24 hours of symptom onset according to standard clinical guidelines. NIHSS (including level of consciousness, gaze, visual fields, facial palsy, motor strength, limb ataxia, sensory deficit, dysarthria, and amnesia) were assessed in both groups at baseline, 1, 2, and 3 months after the intervention.

All patients were monitored for potential adverse events related to metformin, including gastrointestinal symptoms (nausea, vomiting, diarrhea), hypoglycemia, or lactic acidosis. Any adverse event observed was recorded and managed according to clinical guidelines.

### Data Analysis

Data were analyzed using SPSS v.19 software (IBM Corp., Armonk, NY, USA) at a significance level of less than 0.05.

### Ethical Considerations

All the patients were assured that their information would remain confidential.

## Results

**Figure-1:**
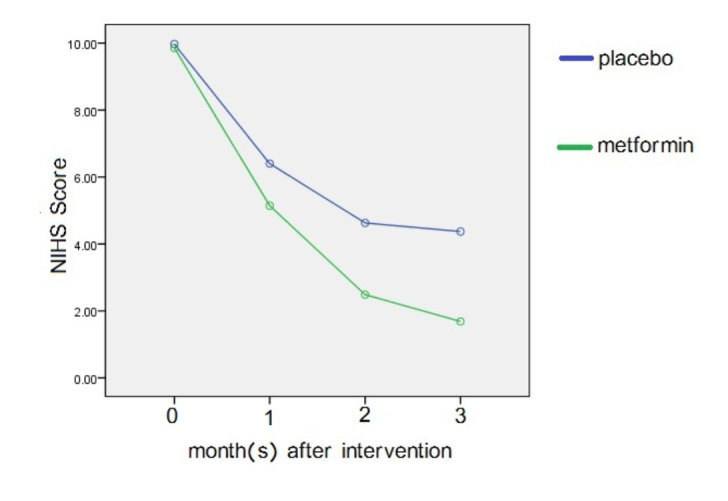
Figure 1. Comparison of NIHSS at baseline, one, two and three months after the intervention

**Table:** Table1. Comparison of Mean and SD of Age and Gender in the Metformin and Placebo Groups

	Metformin	Placebo	P value
Age	70.85±8.60	69.88±7.54	0.494
	N(P)	N(P)	P value
Male	18(51.42%)	20(57.14%)	0.405
Female	17(48.57%)	15(42.85%)	**-**

**Table:** Table2. Comparison of the Mean and SD of NIHSS at Baseline, One, Two and Three Months after the Intervention in the Metformin and Placebo Groups

**Timepoint**	**Metformin (n)**	**NIHSS Mean** **±** **SD**	**Placebo (n)**	**NIHSS Mean** **±** **SD**	**P value**
Baseline	35	9.85±5.74	35	9.97±6.79	0.666
One month	35	5.14±4.74	35	6.40±5.20	0.295
Two month	35	2.48±2.93	35	4.62±4.31	0.021
Three month	35	1.68±2.34	35	4.37±4.00	0.003

**Table:** Table3. Comparison of the Mean and SD of Visual Field and Facial Palsy at Baseline, One, Two, and Three Months after Intervention

Visual field	Metformin (n)	Visual field Mean±SD	Placebo (n)	Visual field Mean±SD	P value
Baseline	35	0.085±0.284	35	0.285±0.572	0.0001
One month	35	0.057±0.235	35	0.200±0.472	0.001
Two month	35	00.00±00.00	35	0.200±0.472	0.0001
Three month	35	00.00±00.00	35	0.200±0.472	0.0001
					
Facial palsy	Metformin (n)	Visual field Mean±SD	Placebo (n)	Visual field Mean±SD	P value
Baseline	35	1.114±0.718	35	1.057±0.838	0.569
One month	35	0.571±0698	35	0.714±0.667	0.389
Two month	35	0.228±0.490	35	0.517±0.654	0.003
Three month	35	0.200±0.405	35	0.517±0.654	0.0001
					
Timepoint	Metformin (n)	Visual field Mean±SD	Placebo (n)	Visual field Mean±SD	P value
Baseline	35	0.142±0.355	35	0.142±0.355	≥0.05
One month	35	0.0286±0.169	35	0.0286±0.169	≥0.05
Two month	35	00.00±00.00	35	00.00±00.00	≥0.05
Three month	35	00.00±00.00	35	00.00±00.00	≥0.05

**Table:** Table4. Comparison of the Mean and SD of Motor Strength and Level of Consciousness at Baseline, One, Two, and Three Months after the Intervention

Arms strength	Metformin (n)	Arms Strength Mean±SD	Placebo (n)	Arms Strength Mean±SD	P value
Baseline	35	2.00±0.939	35	1.800±0.900	0.367
One month	35	1.257±0.885	35	1.514±0.742	0.193
Two month	35	0.600±0.650	35	1.114±0.676	0.002
Three month	35	0.428±0.608	35	1.057±0.639	0.0001
					
Legs strength	Metformin (n)	Arms Strength Mean±SD	Placebo (n)	Arms Strength Mean±SD	P value
Baseline	35	2.228±1.00	35	2.08±0.950	0.543
One month	35	1.457±1.010	35	1.542±0.816	0.697
Two month	35	0.942±0.872	35	1.114±0.718	0.373
Three month	35	0.458±0.658	35	1.057±0.683	0.001
					
Level of consciousness	Metformin (n)	Arms Strength Mean±SD	Placebo (n)	Arms Strength Mean±SD	P value
Baseline	35	0.657±0.683	35	0.942±0.937	0.150
One month	35	0.257±0.443	35	0.400±0.650	0.287
Two month	35	0.057±0.235	35	0.171±0.382	0.137
Three month	35	0.028±0.169	35	0.171±0.382	0.047

**Table:** Table5. Comparison of the Mean and SD of Level of Consciousness based on the Ability of Patients to Obey Verbal Commands at Baseline, One, Two, and Three Months after the Intervention

Level of consciousness	Metformin (n)	Level of Consciousness Mean±SD	Placebo	Level of Consciousness Mean±SD	P value
Baseline	35	0.826±0.821	35	0.742±0.816	0.663
One month	35	0.228±0.490	35	0.257±0.505	0.811
Two month	35	0.057±0.235	35	0.142±0.355	0.016
Three month	35	0.057±0.235	35	0.085±0.284	0.648

**Table:** Table6. Comparison of Mean and SD of Sensory Deficit and Dysarthria at Baseline, One, Two and Three Months after Intervention

Sensory deficit	Metformin (n)	Sensory Deficit Mean±SD	Placebo (n)	Sensory Deficit Mean±SD	P value
Baseline		0.371±0.598	35	0.628±0.689	0.163
One month		0.285±0.518	35	0.485±0.658	0.025
Two month		0.228±0.426	35	0.457±0.610	0.001
Three month		0.228±0.426	35	0.428±0.608	0.003
					
Dysarthria	Metformin (n)	Sensory Deficit Mean±SD	Placebo (n)	Sensory Deficit Mean±SD	P value
Baseline	35	0.685 ±0.718	35	0.628±0.877	0.364
One month	35	0.200±0.472	35	0.428±0.698	0.003
Two month	35	0.028±0.169	35	0.371±0.645	0.0001
Three month	35	0.028±0.169	35	0.371±0.645	0.0001

In this study, 70 patients were randomly divided into two groups of 35. The mean age of the patients in the metformin and placebo groups was estimated to be 70.85±8.60 and 69.88±7.54, respectively. Also, 18 (51.42%) and 20 (57.14%) patients in the metformin and placebo groups were men, respectively. Also, no significant difference was observed between the two groups regarding age and gender (Table-1).

There was no significant difference between the two groups regarding NIHSS at baseline and one month after the intervention (P=0.666 and P=0.295). However, two and three months after the intervention, the metformin group showed a greater decrease, indicating an improvement in neurological function (P=0.021 and P=0.003). The results showed a statistically significant (Table-2, Figure-1) difference between two groups regarding visual field at baseline, one, two, and three months after the intervention (P≤0.05); So that in the intervention group, the mean reached zero after two and three months.

Although baseline Visual Field scores differed between groups, adjusted analyses using ANCOVA confirmed that the improvement observed in the metformin group at 1, 2, and 3 months remained statistically significant (p≤0.05). Also, no significant difference was observed between the two groups at baseline and one month after the intervention regarding facial palsy (P=0.569 and P=0.389). However, two and three months after the intervention, the metformin group showed a greater decrease in facial palsy, indicating improved facial muscle function (P≤0.05, Table-3).

On the other hand, two and three months after the intervention, the mean score of motor strength in the metformin group was significantly lower than the placebo group (P≤0.05). However, no significant difference was observed between the two groups regarding the level of consciousness and the level of consciousness based on the (Table-4) patient's ability to respond.

Most patients had normal gaze at all time points; therefore, mean values and SDs were identical between the metformin and placebo groups, reflecting the absence of gaze abnormalities rather than a data error. Based on the results, two months after the intervention in the metformin group, the mean score of the level of consciousness based on the ability of the patients to respond was lower than in the placebo group. Statistical analyses showed a significant relationship between the level of consciousness based on the ability of the patients to respond and the use of metformin in patients with ischemic stroke (P≤0.05, Table-5). In terms of sensory deficit and dysarthria over time, the mean score of the metformin group significantly decreased compared to the placebo group (P≤0.05). During the study period, no serious adverse (Table-6) events were observed in either group. Mild gastrointestinal symptoms, including nausea and diarrhea, occurred in 2 patients (5.7%) in the metformin group and resolved spontaneously without any intervention. No instances of hypoglycemia or lactic acidosis were reported.

All randomized patients completed the follow-up assessments, and no missing data were observed for any outcome measure.

## Discussion

The present study was conducted to investigate the effect of metformin on the clinical course of non-diabetic patients with ischemic stroke. In this study, 70 patients were randomly divided into metformin and placebo groups. The results showed that metformin has a positive effect on some clinical aspects of the patients. In this study, due to the lack of statistically significant differences in baseline variables such as age and gender between the metformin and placebo groups, the homogeneity of the groups was ensured and it was possible to examine the net effect of metformin. Metformin, with its positive effect on angiogenesis and increased AMPK and antioxidant effects, has shown acceptable results in improving the symptoms of ischemic stroke in early studies on rats and diabetic patients who have used this drug chronically [13]. In a study by Sarkaki et al., improvement in neurological, anxiety, and motor symptoms was observed [14].

We have arrived at conclusions aligned with preceding RCTs and preclinical studies, confirming the neuroprotective effects of metformin in stroke patients without diabetes. The reference to clinical studies and studies on animals allows us to build a stronger evidence-based argument on the gains made in the motor, sensory, and visual domains. This coherence with the underlying research augments the justification of our findings and underscores the potential of metformin for clinical application in stroke recovery.

In a review study by Jia and Cheng, it was shown that acute metformin administration in non-diabetic rats could have a favorable effect on the prognosis of patients with ischemic stroke. In this study, it was found that the use of metformin after ischemic stroke has no effect on acute cerebral infarction, but it improves cerebral AMPK, neural function, the growth of microglial cells and macrophages, angiogenesis, and neurogenesis in the ischemic focus [15]. Also, in a study by Abbasi et al., metformin consumption played a role in reducing the NIHSS of non-diabetic stroke patients.

Several randomized controlled trials in non-diabetic stroke patients have investigated the neuroprotective effects of metformin. For instance, Abbasi et al. (2018) reported significant reductions in NIHSS scores over a three-month follow-up period. Collectively, these studies provide supporting evidence for the potential of metformin as an adjunct therapy to improve neurological outcomes in non-diabetic patients with ischemic stroke.

Based on the results of the study, in cortical stroke, the difference in NIHSS between the case and control groups was not significant on the first, third, and seventh days of the study. However, a statistically significant difference was observed between the case and control groups regarding the NIHSS during the first, second, and third months [16].

The results of our study showed that there is no significant difference between the two groups regarding the NIHSS at baseline and one month after the intervention; however, two and three months after the intervention, the group receiving metformin had a significant decrease in NIHSS (P=0.021 and P=0.003, respectively). By categorizing symptom scores, we accounted for the heterogeneity of stroke manifestations among patients, providing a more precise evaluation of metformin’s effect on each neurological domain. These results suggest that the beneficial effects of metformin in improving neurological function in ischemic patients appear more in the subacute or subacute phase of the disease and may play an important role in neurological recovery and repair after ischemic injury [17].

On the other hand, in the assessment of visual function, the metformin group had a significant improvement compared to the control group at all follow-up times (P=0.001), which is consistent with the results of a study by Chen et al. [18]. Improving visual function is one of the important rehabilitation indicators of stroke patients, which plays a vital role in quality of life. Also, in the assessment of facial palsy two and three months after the intervention, the metformin group had a significant decrease in the palsy score. These results indicate that metformin may help improve patients' motor function by enhancing neurogenesis and neural plasticity, which is consistent with the results of a study by Chamorro et al. [19].

However, the level of consciousness of patients in the two groups did not show a significant difference, except three months after the intervention (P=0.047). This suggests that metformin has a more limited effect on general consciousness and that its effects are probably more manifested in motor and sensory aspects. Related studies on the cognitive effects of metformin after stroke are still limited and require more extensive studies. Sensory deficits and dysarthria also improved significantly in the metformin group (p≤0.05), which is consistent with the anti-inflammatory and neurotrophic factor-stimulating mechanisms reported in previous studies [20][21]. The improvement in these indicators indicates the broad effects of metformin in improving neurological damage after stroke.

Overall, the results of this study showed that metformin administration in non-diabetic patients with ischemic stroke significantly improved clinical recovery, especially regarding motor, sensory, and visual function. Since metformin is a cost-effective drug with a relatively high safety profile, its use as an adjunct therapy in the rehabilitation of stroke patients can be very useful. However, generalization of these results requires multicenter studies with larger sample sizes and longer follow-up periods in order to more confidently state the role of metformin in improving clinical outcomes in stroke patients.

## Conclusion

Based on the results, metformin at a dose of 500 mg daily for three months in non-diabetic patients with acute ischemic stroke had a very favorable effect on improving the symptoms of these patients, and the greatest effect of the drug was two and three months after the intervention.

## Conflict of Interest

The authors declare that they have no conflicts of interest.
